# Case Report: Borderline tumor and primary peritoneal carcinoma - a rare synchronism

**DOI:** 10.12688/f1000research.20420.2

**Published:** 2019-11-08

**Authors:** Mariana Rei, Sofia Raposo, Paulo Figueiredo, Rita Sousa, Luís Sá

**Affiliations:** 1 Department of Obstetrics and Gynecology, Centro Hospitalar Universitário São João, Porto, Porto, 4200-319, Portugal; 2 Unit of Obstetrics and Gynecology, Faculty of Medicine, University of Porto, Porto, Portugal; 3 Department of Gynecology, Instituto Português de Oncologia Francisco Gentil de Coimbra, Coimbra, Portugal; 4 Department of Pathology, Instituto Português de Oncologia Francisco Gentil de Coimbra, Coimbra, Portugal

**Keywords:** borderline tumor, ovarian neoplasm, peritoneal neoplasm, high grade serous carcinoma, low grade serous carcinoma

## Abstract

Ovarian borderline serous tumors present with peritoneal involvement in 20% of cases, either as non-invasive or invasive implants, the latter also known as extraovarian low-grade serous carcinoma. The coexistence of high-grade serous carcinoma is rare, suggesting a synchronous neoplasia with a distinct and independent tumor biology and behavior. We aim to describe a case of a synchronous ovary-peritoneum neoplasia: serous borderline tumor and primary peritoneal high-grade serous carcinoma. A discussion and literature review concerning the optimal diagnostic and therapeutic approach is provided.

## Introduction

Serous borderline tumors/atypical proliferative serous tumors (SBT/APST) are defined as non-invasive tumors displaying greater epithelial proliferation and cytological atypia than their benign counterparts. However, the SBT/APST show less cellular atypia than low-grade serous carcinoma (LGSC) ^1^. This entity is considered the premalignant lesion of LGSC and usually occurs in women 10 to 15 years younger than those with serous carcinoma. Although there are some similarities in risk-factor associations to high-grade serous carcinoma (HGSC), infertility is more common and there is usually no relation with *BRCA1*/ *BRCA2* mutations ^2, 3^.

Peritoneal lesions associated with SBT/APST may occur in 20% of cases and were classically defined as non-invasive or invasive implants based on the capacity to infiltrate the underlying tissue. The non-invasive implants are further subdivided into desmoplasic or epithelial types and have almost no negative influence on the 10-year survival rates. Distinctively, the invasive form behaves like LGSC, featuring a poor prognosis with a 50% recurrence rate and a 35% 10-year survival rate ^2, 4, 5^.

Therefore, the morphology of the peritoneal implants is the main prognostic factor for patients presenting with stage II-III SBT/APST ^1, 5^. However, implants are heterogeneous, different histologic patterns may coexist, and unequivocal invasion may be difficult to establish in some cases. A comprehensive histopathological examination of multiple fragments of peritoneal implants is recommended in order to optimize the differential diagnosis between non-invasive and invasive implants.

Primary peritoneal carcinoma (PPC) resemble low- or high-grade serous ovarian counterpart and occurs in women with a mean age of 62 years. The estimated lifetime risk is 1 per 500 women, and nearly 15% of common epithelial ovarian cancers are in fact PPC ^1^. Histology and immunohistochemistry (IHC) are virtually indistinguishable from epithelial ovarian carcinoma and the most common histological variant is HGSC, although other histologic types have also been reported. In order to meet criteria for PPC, both ovaries and tubes should be macro- and microscopically normal ^1^. From a clinical point of view, the clarification of the tumor origin may not be critical, since the oncologic behavior, treatment and prognosis largely overlap their tubal and ovarian counterparts and therefore are addressed similarly. Conversely, the distinction between LGSC and HGSC is of utmost importance concerning diagnostic, therapeutic approaches and prognosis.

The coexistence of SBT/APST and HGSC is rare, suggesting a synchronous neoplasia with a distinct and independent tumor biology and behavior. We aim to describe a case of a synchronous ovary-peritoneum neoplasia. A discussion and literature review concerning the optimal diagnostic and therapeutic approach is provided.

## Case report

We present the case of a 52-year-old postmenopausal woman with no relevant medical history, referred to an oncologic center due to a voluminous adnexal mass. She clinically presented with metrorrhagia, asthenia and anorexia with a significant weight loss. Serum tumor markers Ca125, HE4 and Ca 72.4 were significantly increased (1058 U/mL, 1795 pmol/L and 9.5 U/mL, respectively). Pelvic ultrasonography revealed a large heterogeneous multicystic adnexal mass, with multiple papillae and irregular intern contour. Thoraco-abdominal-pelvic computerized tomography (CT) scan revealed a vascularized heterogeneous adnexal mass measuring 190×100 mm; no ascites or unequivocal signs of peritoneal carcinomatosis or distant dissemination were observed. The validated preoperative diagnosis models ROMA, LR2 and ADNEX were calculated, presenting, respectively, a 34.8%, 72.8% and 85.8% risk of malignancy ^6– 9^.

Within two weeks, the case was discussed in the multidisciplinary gynecologic oncology tumor board, advising for laparotomy with frozen section of the suspicious lesions. The patient was then submitted to exploratory laparotomy, revealing a frozen pelvis and peritoneal carcinomatosis: the right ovary was transformed into a voluminous neoplasia; independently, a large tumor mass involving omentum and the transverse portion of colon, apparently not surgically resectable; the left ovary and both fallopian tubes were macroscopically normal. Intraoperative frozen section of the right ovary revealed a borderline tumor, whereby cytoreductive surgery was performed, including hysterectomy, double adnexectomy, omentectomy and resection of the peritoneal implants. The final cytoreduction was complete for the pelvis but incomplete for the superior abdomen, with over 2 cm of residual disease (R2).

**Figure 1.**
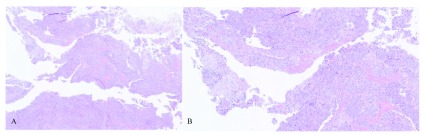
Histologic examination of primary peritoneal high-grade serous carcinoma (HGSC). Hematoxylin and eosin (H&E) staining, original magnification 1A:X4; 1B:X10.

The final histology with hematoxylin and eosin staining revealed two synchronous tumors: SBT of the right ovary and HGSC of probably primary peritoneal origin, FIGO stage IIIC ( Figure 1A–B and Figure 3A–B). Multiple tissue blocks from the ovarian tumor were examined in the microscopy exam and none identified invasive component. The left ovary did not show any signs of either malignancies. Both fallopian tubes were analyzed according to the Sectioning and Extensively Examining the Fimbriated End Protocol (SEE-FIM) and no precursor lesions in the fallopian tube epithelia were found. Additionally, no lesions were found in the hysterectomy specimen.

Morphologically, the peritoneal implants displayed branching papillary fronds, slit-like fenestrations, glandular complexity, moderate to marked nuclear atypia with marked pleomorphism, prominent nucleoli, stratification, frequent mitoses and stromal invasion (irregular or destructive infiltration by small glands or sheets of cells), strongly indicating HGSC ( Figure 1A–B). Conversely, features suggestive of SBT/APTS were found in the right ovary, namely broad, branching papillae (hierarchical branching) focally covered by stratified epithelium with mild to moderate atypia with few mitoses ( Figure 3A–B).

**Figure 2.**
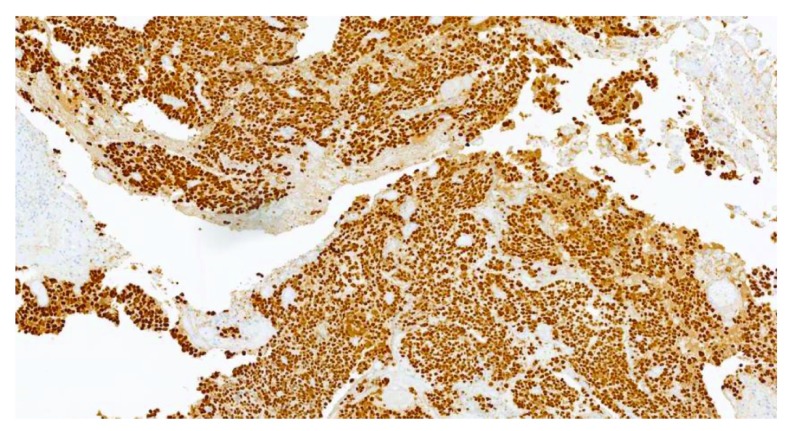
Strong and diffuse immunoexpression of p53 in primary peritoneal high-grade serous carcinoma (HGSC), original magnification X10.

**Figure 3.**
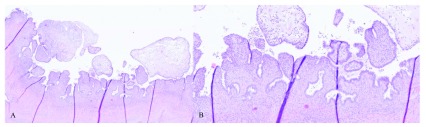
Histologic examination of serous borderline tumor (SBT). Hematoxylin and eosin (H&E) staining, original magnification 3A: X4; 3B:X10.

**Figure 4.**
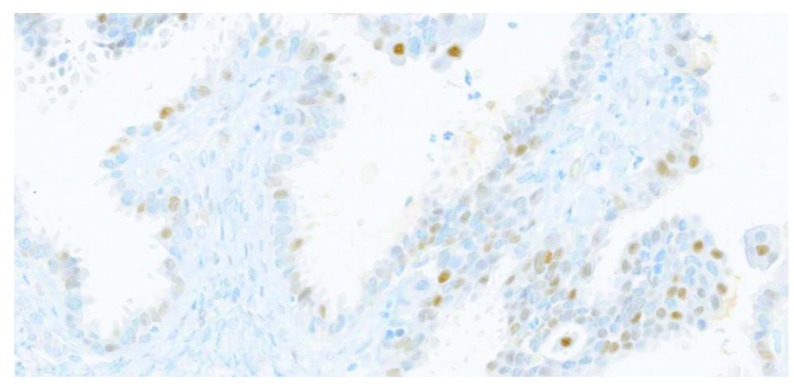
Wild-type immunoexpression of p53 in serous borderline tumor (SBT), original magnification X40.

By IHC, the HGSC specimen showed positivity for PAX8, WT-1, aberrant p53 expression and high proliferation index, while calretinin and CD10 staining were negative ( Figure 1 and Figure 2). Estrogen but not progesterone receptor positivity was found in HGSC. In contrast, SBT specimen was characterized by expression of WT1 and PAX8 and both estrogen and progesterone receptors, and p53 was wild-type ( Figure 3 and Figure 4). Molecular genetic analysis was not performed in any of the specimens.

The post-operative CT scan performed four weeks later revealed rapid peritoneal disease progression, with high-volume ascites and hydronephrosis, resulting in an important decline on clinical status and death before initiating palliative chemotherapy.

## Discussion

The dualistic model of ovarian carcinogenesis recognizes two distinct categories in epithelial ovarian carcinomas: the more common aggressive type II tumors and the less common slow-growing type I tumors ^10^. A revised model takes into account the current histopathologic classification, providing a bridge into the future by integrating emerging molecular genetic findings ^5^. Type I tumors arise mostly from borderline tumors and include endometriosis-related tumors, LGSC, mucinous carcinomas and malignant Brenner tumors. Distinctively, type II tumors seem to develop from tubal intraepithelial carcinomas (STICs) that disseminate as carcinomas involving ovary and extraovarian sites, in most cases corresponding to HGSC. Moreover, Malpica et al evaluated a two-tier system which subdivides serous carcinomas into low- and high-grade, implying independent pathogenesis and displaying distinct morphology, IHC and molecular biology ^2, 5, 11^.

Histopathologically, SBTs are characterized by a hierarchical papillary or micropapillary pattern, low-grade nuclear atypia and low mitotic activity. Most SBTs and LGSCs show PAX2 expression but no aberrant p53 expression and p16 overexpression. The molecular genetic analysis frequently demonstrates *KRAS* or *BRAF* mutation but *TP53* mutation is rare. In contrast, HGSCs present an invasive growth pattern, high-grade nuclear atypia and intense mitotic activity. Aberrant p53 expression, p16 overexpression and high MIB-1 labeling index are common; molecular studies frequently reveal *TP53* mutation, but *KRAS*/ *BRAF* mutation is infrequently reported ^12, 13^.

Nevertheless, it is still not clear whether some type II tumors develop from type I tumors. Dehari *et al.* ^14^ studied the clonality of six cases of HGSC closely related to SBT/APST and invasive micropapillary LGSC from a cohort of 210 ovarian serous tumors. A morphologic continuum between the high-grade and the low-grade tumors was observed in four cases; the same KRAS mutations were found in both the SBT/APST and HGSC component of the tumor, indicating a clonal relationship and suggesting that in rare cases, HGSC may arise from SBT/APST ^14^.

We report a case of a rare synchronism of neoplasms with distinct carcinogenesis pathways: SBT/APST of the right ovary with preserved contralateral ovary and HGSC identified in peritoneal implants. Peritoneal involvement could be related to multiple origins: non-invasive implants of SBT/APST, invasive implants or extraovarian LGSC, coexistence of other ovarian or secondary malignancy. An accurate histopathological and immunohistochemical analysis is of utmost importance in order to better delineate treatment and prognosis.

In this case, the ovarian tumor showed positivity for PAX8 and WT1. PAX8 is expressed in ovarian and endometrial neoplasias, whereas WT1 is expressed in ovarian serous tumors and is useful to support ovarian origin. Estrogen receptor expression was also positive. On the other hand, both progesterone receptors and CD10 were negative by IHC analysis.

*TP53* mutations integrate the molecular carcinogenesis pathway of STIC and are ubiquitous in HGSC, therefore optimizing the differential diagnosis between HGSC and LGSC. p53 is widely used as a surrogate for *TP53* mutation; a recent study showed that it can approach 100% specificity for *TP53* mutation, and its high negative predictive value is clinically useful to exclude the possibility of a LGSC ^2, 12^. In the current report, peritoneal implants stained positive for this marker, excluding the diagnosis of non-invasive implants of SBT/APST or LGSC and reinforcing the coexistence of HGSC. Identification of HGSC in peritoneal implants with a preserved contralateral ovary and tube strongly suggests a primary peritoneal origin.

The diagnosis of peritoneal carcinomatosis related to HGSC majorly impacts therapeutic approach. While there is no evidence to recommend neoadjuvant or adjuvant chemotherapy in SBT/APST, the diagnosis of HGSC with a high peritoneal cancer index (PCI) might have altered the course of the surgical approach in favor of chemical cytoreduction with neoadjuvant chemotherapy.

Nearly 15 to 20% of HGSC are associated with *BRCA1*/ *BRCA2* germline mutations ^15^. Its presence impacts therapy and prognosis, since these tumors are highly sensitive to poly (ADP-ribose) polymerase inhibitors, showing improved survival comparing to non-carriers ^13^. Hence, there is now strong evidence to recommend genetic testing to all women affected with high-grade epithelial non-mucinous ovarian/tubal/peritoneal cancer ^15^.

The diagnosis of a borderline tumor with peritoneal implants imposes the challenging differential diagnosis between non-invasive implants, LGSC and the rare coexistence of HGSC of ovarian or extraovarian origin. This last entity presents a distinct tumor biology and carcinogenesis profile, with strong implications in therapeutic approach and prognosis. Whenever unresectable disease is strongly suspected, adnexectomy or ovarian biopsy in association with peritoneal implants biopsy would allow an accurate diagnosis of a synchronous neoplasia. A peritoneal disease with high tumor burden due to HGSC could impact the optimal therapeutic approach, namely the possibility of neoadjuvant chemotherapy and interval surgery. The discrepancy between tumor markers, imaging criteria and malignancy scores should raise the clinical suspicion for the coexistence of a synchronous neoplasia with a more aggressive oncologic behavior.

## Data availability

All data underlying the results are available as part of the article and no additional source data are required.

## Consent

Written informed consent for publication of clinical details and images was obtained from a relative of the patient.
