# Supplementary material for: Case Report: Borderline tumor and primary peritoneal carcinoma - a rare synchronism
Source: F1000Res. 2019 Nov 8;8:1630. Originally published 2019 Sep 12. [Version 2] doi: 10.12688/f1000research.20420.2 (PMC6871352; doi:10.12688/f1000research.20420.2)
Supplement: Peer review history

# Peer review: Reviewer response for version 2

**DOI:** 10.5256/f1000research.23403.r56356

**Published:** 2019-11-18

**Authors:** Carla Bartosch

**Affiliations:** 1 Department of Pathology, Portuguese Oncology Institute of Porto, Porto, Portugal

The authors have much improved the manuscript.

 I only further suggest using a higher power magnification for figures 1B and 3B, since the 10x does not provide different information compared to the 4x.

I confirm that I have read this submission and believe that I have an appropriate level of expertise to confirm that it is of an acceptable scientific standard.

# Peer review: Reviewer response for version 1

**DOI:** 10.5256/f1000research.22444.r54372

**Published:** 2019-10-18

**Authors:** Maria Dolores Diestro

**Affiliations:** 1 Gynecologic Oncology Unit, IdiPAZ (Hospital La Paz Institute for Health Research), La Paz University Hospital, Madrid, Spain

From the point of view and optics of Oncological Gynaecology, it would highlight the originality of the clinical case presented.

 The presentation of clinical and surgical data is adequate and meticulous and translates a good level of background knowledge of the author.

 Good writing, clarity in the presentation and good structuring of the discursive dialogue of the discussion.

 Perhaps there would be a short synthesis at the end of the article with the background of the case and a small conclusion.

I confirm that I have read this submission and believe that I have an appropriate level of expertise to confirm that it is of an acceptable scientific standard.

# Response

**Published:** 2019-11-05

**Authors:** Mariana Rei

**Affiliations:** Centro Hospitalar Universitário São João, Portugal

Thank you for your kind reply and consideration of the manuscript “Borderline tumor and primary peritoneal carcinoma: a rare synchronism” for revision. The reviewers’ comments were highly appreciated and were thoroughly taken into consideration.

1.Additional clinical details were provided in order to demonstrate the pathological diagnosis in which the case relies on, namely regarding the morphological and immunohistochemical features and a new set of histologic images.

2.In the last paragraph of the Discussion section we display a final summary of the main findings and their implications for the clinical practice, while trying to be clear and concise.

Thank you in advance for your encouraging and inspiring review. We hope that the manuscript is now appropriate for being considered for publication. Please do not hesitate to contact me if any further information is needed.

Kind regards,

Mariana Rei, MD

# Peer review: Reviewer response for version 1

**DOI:** 10.5256/f1000research.22444.r53847

**Published:** 2019-10-14

**Authors:** Carla Bartosch

**Affiliations:** 1 Department of Pathology, Portuguese Oncology Institute of Porto, Porto, Portugal

This is an interesting case report, presenting the rare finding of a serous borderline ovarian tumor with a synchronous high grade serous carcinoma in the peritoneum. The introduction and discussion are well written. The case report itself, essentially relies on the pathological diagnosis, which in my opinion is insufficiently demonstrated.

 My comments are remarks are the following:

**Abstract:**

- “either as non-invasive or invasive implants, also known as extraovarian low-grade serous carcinoma.” – change to “the latter also known…”

**Introduction:**

- “In order to meet criteria for PPC, both ovaries and tubes should be macro- and microscopically normal in size or enlarged by a benign process” – This is not entirely correct. If the tube has, for example, STIC, the tumor should be considered of tubal origin, yet it may have a perfectly normal size.
- “largely overlap their ovarian counterparts” – change to “tubal and ovarian counterparts”

**Case report:**

- The ovarian tumor is large (19cm), was an adequate sampling done to exclude an invasive component?
- In the presence of a HGSC in the peritoneum, total inclusion of the adnexa, particularly the tube following the SEE-FIM protocol, should be done to exclude a tubal primary – was this done? Were there any precursor lesions in the Fallopian tube epithelia? The case reports a “frozen pelvis” – weren’t the tubes involved by the tumor?
- HGSC can architecturally simulate LGSC in the peritoneum. The two basic criteria to distinguished HGSC and LGSC are the nuclear atypia and mitotic index (as emphasized in manuscript reference 11), but none of which are mentioned in the case description.
- HGSC may also simulate a BST architecture in the ovary, but the cytological features and immunohistochemical profile are very different. Please provide the morphological description (including architectural patterns, cytological features, mitotic index) as well as the immunohistochemical profile of both neoplasias to support the diagnosis.
- p53 should be report as wild type or aberrant (null or diffusely positive). Variable p53 positivity occurs in wild type patterns. Aberrant p16 overexpression and a very high proliferation index (Ki-67) would also support the differential diagnosis between HGSC and LGSC.
- Were there any lesions in the hysterectomy specimen, namely in the endometrium?
- Histological pictures are all low-power. A and C show similar aspects. Legend mentions the left ovary, which is not shown – consider deleting this from the legend as it is already in the case report text. I suggest making a panel with low and high power magnifications showing the architectural and cytological features, plus p53 expression, of both neoplasias.
- Was any genetic study performed, namely for BRCA1/2 or BRAF mutations?

**Discussion:**

- First paragraph – Kurman’s dualistic model and Malpica’s two tier system are different concepts. Thus, the last phrase can not start with “this”.
- Estrogen and progesterone expression is mentioned in the discussion, but is not in the case report description. Provide the extension of ER and PR expression in both tumors. PR expression was negative in the BST?

I confirm that I have read this submission and believe that I have an appropriate level of expertise to confirm that it is of an acceptable scientific standard, however I have significant reservations, as outlined above.

# Response

**Published:** 2019-11-05

**Authors:** Mariana Rei

**Affiliations:** Centro Hospitalar Universitário São João, Portugal

Reply to Reviewers

         Thank you for your kind reply and consideration of the manuscript “Borderline tumor and primary peritoneal carcinoma: a rare synchronism” for revision. Your comments were highly appreciated and were thoroughly taken into consideration, as following: 

1. Additional clinical details were provided in order to demonstrate the pathological diagnosis in which the case relies on.

2. Regarding the diagnosis of the SBT in the right ovary,multiple tissue blocks from the ovarian tumor were examined in the microscopy exam and none identified invasive component. Both adnexa weretotally included andanalyzed according to the Sectioning and Extensively Examining the Fimbriated End Protocol (SEE-FIM) and noprecursorlesionsin the fallopian tube epithelia were found.

3. More detailed information regarding the morphological and immunohistochemical features of both tumor specimens was provided, allowing to distinguish between SBT and HGSC and to rule out LGSC. Regarding IHC, p16 was not performed in either specimen, therefore we could not provide that data.

4. The molecular genetic analysis was not performed, therefore we could not provide that set of data.

5. The concepts of ovarian tumor carcinogenesis models described in the first paragraph of the discussion, namely Kurman’s dualistic model and Malpica’s to tier system, were further clarified. 

6. Finally, the legend of the histological pictures was modified accordingly and an additional set of images with higher power magnifications plus p53 expression of both tumors was provided. 

NOTE: The major revision work-up is noticed in the third, fourth and fifth paragraphs of the Case Report section and in the first paragraph of the Discussion section. 

Thank you in advance for your encouraging and inspiring review. We hope that the manuscript is now appropriate for being considered for publication. 

Kind regards,

Mariana Rei, MD
